# Rat bite fever mimicking ANCA-associated vasculitis

**DOI:** 10.1007/s00296-023-05369-4

**Published:** 2023-07-14

**Authors:** Aleksandra Błaż, Jan Zalewski, Anna Masiak, Mariusz J. Kujawa, Monika Gosz, Natalia Buda

**Affiliations:** 1 Department of Internal Medicine, Connective Tissue Diseases & Geriatrics, Medical University of Gdańsk and University Clinical Centre in Gdańsk, ul. Dębinki 7, 80-952 Gdańsk, Poland; 2 Division of Radiology, University Clinical Centre in Gdańsk, Gdańsk, Poland

**Keywords:** Fever, Arthritis, ANCA-associated vasculitis, Purpura, Rat bite fever, *Streptobacillus moniliformis*

## Abstract

Rat bite fever (RBF) is a rare infectious zoonotic disease caused by two bacterial species: the Gram-negative rod *Streptobacillus moniliformis* and the Gram-negative coiled rod *Spirillum minus*. The association between RBF and skin vasculitis and arthritis has been observed. The aim of this paper was to present a case of rat-bite fever with symptoms of skin vasculitis and arthritis, associated with high titers of ANCA antibodies and anti-endothelial cell antibodies suggestive of primary vasculitis. The patient was successfully treated with antibiotics and non-steroidal anti-inflammatory drugs, leading to significant improvement. Based on the presented case, we discuss the differential diagnosis of the signs and the role of infection in the induction of ANCA antibodies. We reviewed the English language literature for cases of RBF presenting with symptoms of vasculitis and/or antibody presence. A literature review was performed in PubMed and Google using the keywords “rat bite fever” AND “vasculitis”, “systemic vasculitis”, “ANCA”, “antiendothelial antibodies”. No cases of rat-bite fever with the presence of ANCA antibodies or AECA antibodies in its course have been described thus far. Rat bite fever is a rare disease with nonspecific symptoms. In its course, general weakness, intermittent fever, leukocytoclastic vasculitis, and arthritis are reported. To our knowledge, this is the first reported case of ANCA positivity associated with RBF.

## Introduction

Rat bite fever (RBF) is an uncommon infectious disease that can be contracted from rodents. The disease is caused by two bacterial species, *Streptobacillus moniliformis* and *Spirillum minus*, which are Gram-negative rods typically found in the urine and upper respiratory tract secretions of rodents. [1, 2]. RBF is primarily transmitted through the bite or scratch of infected rodents [1–3], particularly rats, but can also be contracted through ingestion of contaminated food or contact with infected animals. Furthermore, exposure to rodents, even without visible bites or scratches, may pose a risk for transmission. The risk of transmission is estimated to be around 10% [1, 2]. Although rare, domestic animals, such as dogs and cats, can become infected with rat-bite fever and potentially transmit the disease to humans. [1, 2]. It is not spread from one person to another.

Symptoms of RBF caused by *S. moniliformis* are nonspecific. The incubation period typically lasts two or three days, but can occasionally last up to three weeks. Initial symptoms often include general weakness, high fever with chills, headache, vomiting, and myalgia, followed by the appearance of maculopapular rash, purpuric or petechial skin lesions on the distal parts of the limbs. In some cases, histopathological examination of skin lesions reveals leukocytoclastic vasculitis, an inflammation of small blood vessels characterized by the destruction of leukocytes in the vessel walls [2]. These lesions may appear diffusely over the body and may exfoliate in up to 20% of cases. During the course of the disease, 50–70% of patients may develop migratory polyarthralgia and arthritis, which commonly affects the knee, ankle, wrist, elbow, and shoulder joints, as well as small joints of the hands (oligoarthritis has also been described). These symptoms are often accompanied by edema, limited joint mobility, and pain, which may persist for many years (chronic arthropathy has also been reported [4]). Typically, the fever associated with rat-bite fever is prolonged, in some cases lasting for several months and often recurring. Although the condition may be self-limiting, it can also lead to serious complications, including endocarditis (which is sometimes misdiagnosed as rheumatic heart disease [5]), sepsis, liver abscesses or, very rarely, abscesses located in other organs, meningitis, discitis, pneumonia, or acute adrenal cortex insufficiency [1].

Vasculitides refer to a heterogeneous group of medical disorders characterized by inflammatory processes affecting the vascular wall, with primary or secondary underlying causes, such as connective tissue diseases, infections, neoplasms, and medication use. To date, various authors have reported an association between rat-bite fever and the symptoms of vasculitis or arthritis. [5–7].

The aim of this paper is to report a case of rat-bite fever in a patient who exhibited symptoms of skin vasculitis and arthritis, along with high titers of ANCA antibodies and anti-endothelial cell antibodies (AECA) suggestive of primary vasculitis. Upon reviewing the literature, we found no other cases of rat-bite fever associated with the presence of antibodies in high titers. The case presented in this report leads us to discuss the possible role of infection in the induction of ANCA production.

## Case report

**Fig. 1:**
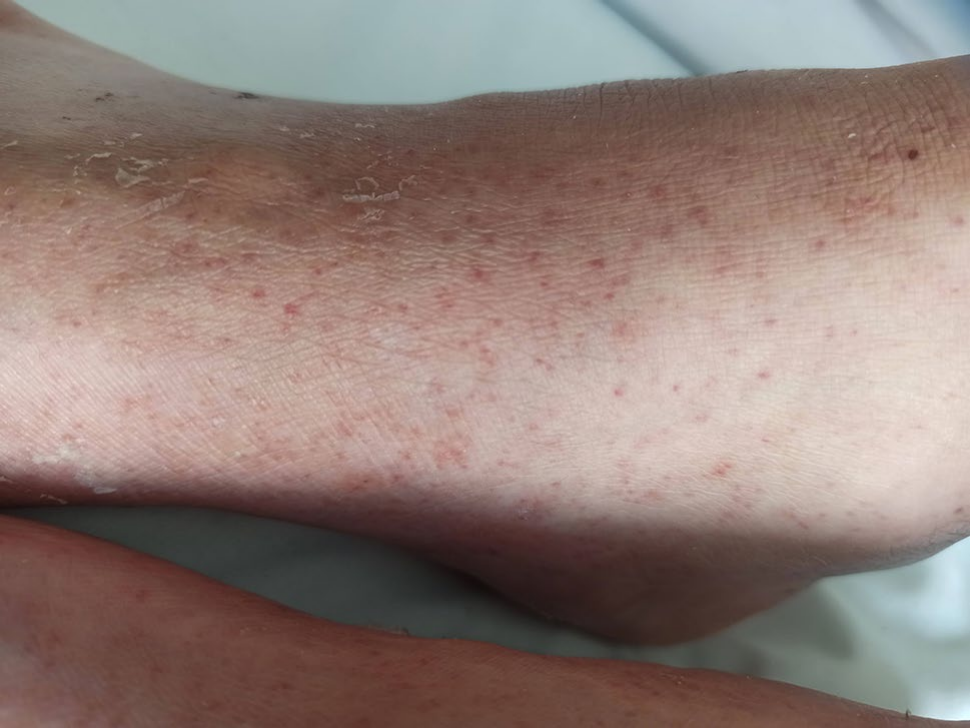
Purpuric lesions present on the feet and legs below the knee

**Fig. 2:**
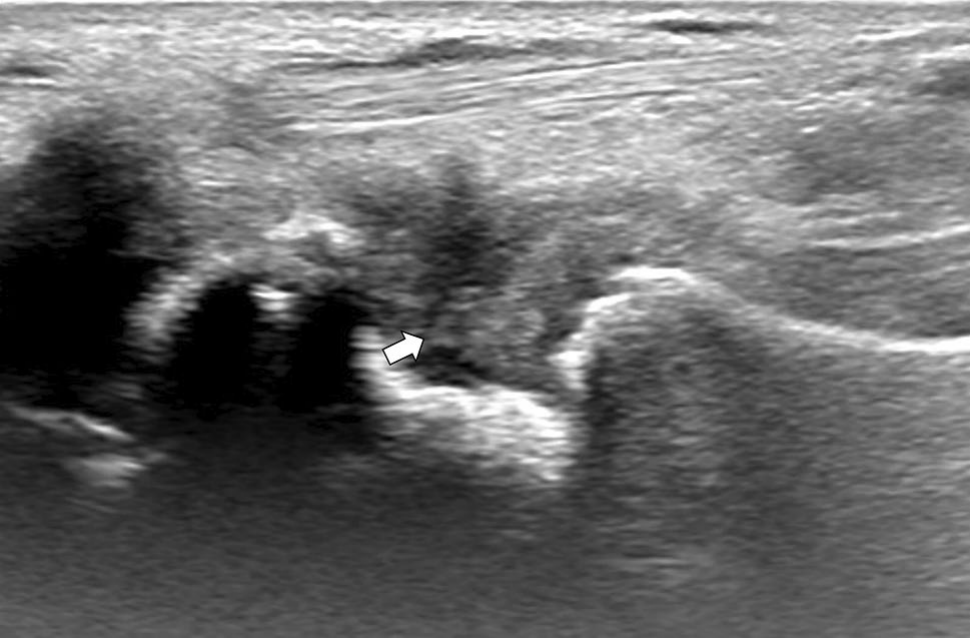
Synovial hypertrophy and joint effusion revealed in ultrasonography of the radiocarpal joint and intercarpal joint on the palmar side

**Fig. 3:**
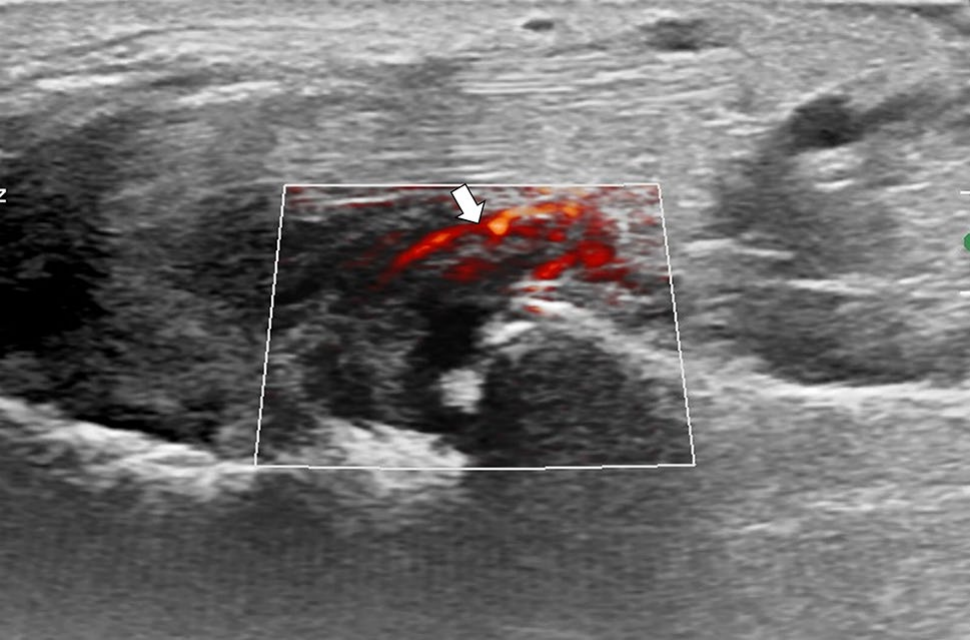
Increased second-degree synovial blood flow revealed in ultrasonography of the radiocarpal joint and intercarpal joint on the palmar side

**Fig. 4:**
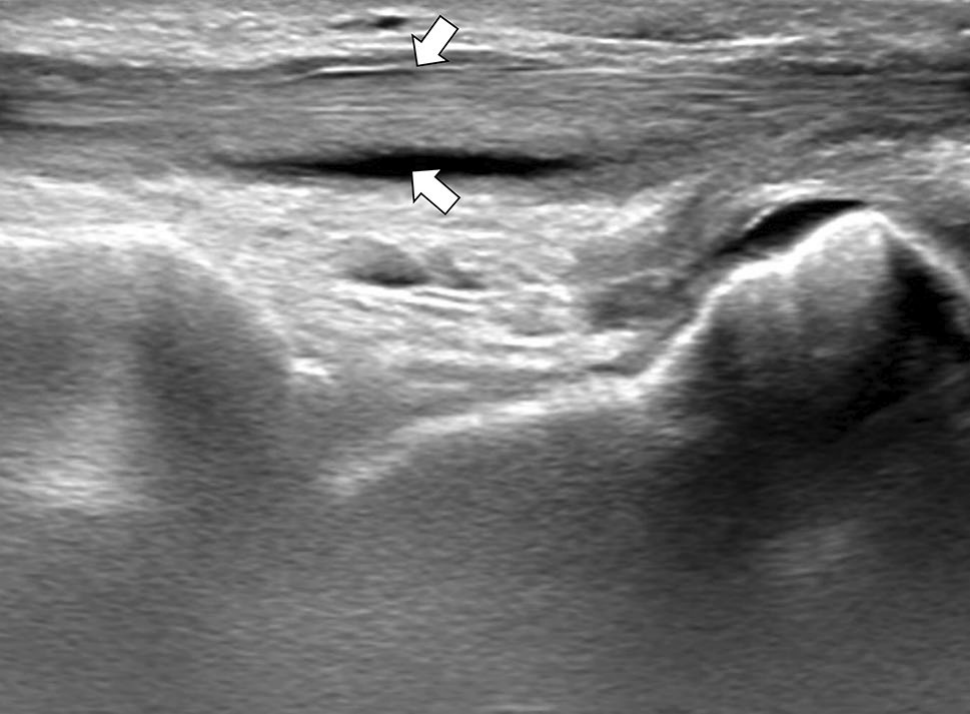
Thickening of tenosynovium, effusion, and synovial hypertrophy revealed in ultrasonography of the left musculus extensor digitorum longus tendon in long axis projection

**Fig. 5:**
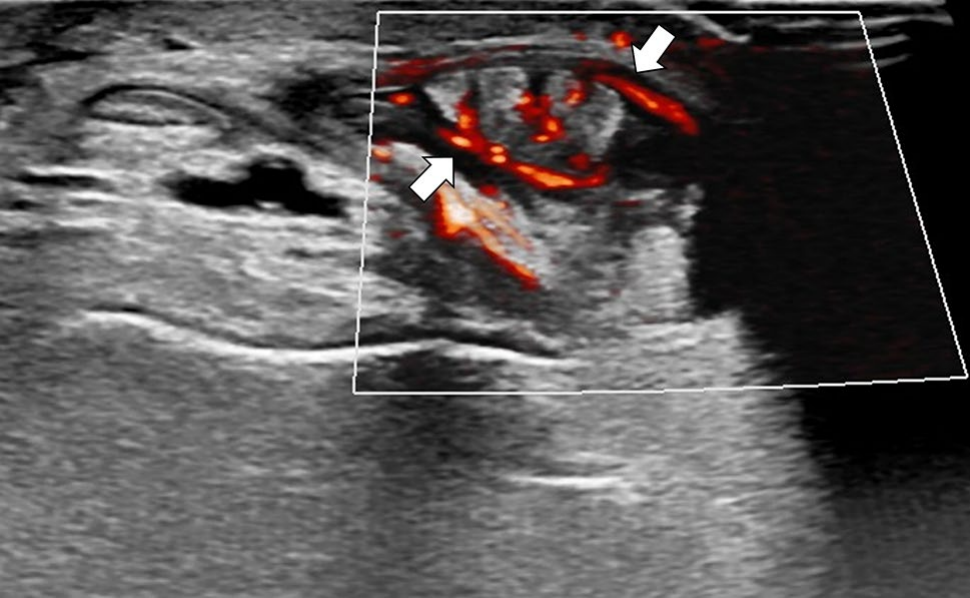
Increased blood flow within the tenosynovium revealed in ultrasonography of the left ankle joint

A 51-year-old male with a history of alcohol addiction, homelessness, and previous employment in rodent extermination, with no known medical comorbidities, was admitted to the hospital with a high fever and suspected sepsis. Upon admission, the patient's general condition was satisfactory, with a conscious mental status, somnolence, psychomotor retardation, and normal circulatory and respiratory function. The physical examination demonstrated notable cutaneous findings, including palpable purpura affecting both lower extremities, and legs below the knee (as illustrated in Fig. 1), accompanied by small ulcerations on the digits suggestive of bites. Additionally, the patient exhibited arthritis and arthralgia involving both wrists and ankles, as well as cervical rigidity without other signs of meningeal irritation, and hepatomegaly. Laboratory tests revealed elevated levels of inflammatory markers, including a C-reactive protein (CRP) level of 265.7 mg/L (normal range 0–5 mg/L), a procalcitonin level of 6.31 ng/mL (normal range < 0.5 ng/mL), and leukocytosis with neutrophilia, indicated by a white blood cell count of 20.5 G/L and a neutrophil count of 19.04 G/L. The liver enzyme levels were mildly elevated, with an aspartate aminotransferase (AST) level of 58 U/L (normal range 5–34 U/L) and a gamma-glutamyl transferase (GGT) level of 188 U/L (normal range 9–36 U/L). However, the alanine aminotransferase (ALT) and alkaline phosphatase (ALP) levels were within the normal range. In addition, the patient had elevated pancreatic enzyme levels, with a lipase level of 283 U/L (normal range 8–78 U/L) and an amylase level of 292 U/L (normal range 25–125 U/L). Given the patient's history of alcohol abuse, these results are suggestive of mild acute pancreatitis. A summary of the laboratory results can be found in Table 1. The meningitis pathogen panel performed with the polymerase chain reaction (PCR) showed negative results. A computed tomography (CT) scan of the chest, abdomen, and pelvis demonstrated hepatomegaly and an indeterminate ileal thickening, while no significant pulmonary abnormalities were observed. Performed transthoracic echocardiography revealed no ultrasonographic symptoms of endocarditis. Synovial hypertrophy with increased blood flow in the synovium and tenosynovium, as well as joint effusions in the wrist and ankle, were detected on joint ultrasonography (refer to Figs. 2, 3, 4, 5). Due to clinical suspicion of systemic disease, the rheumatologic workup was performed and revealed a significantly high titer of ANCA (cANCA/aANCA 1:1280) and AECA (1:2560) antibodies, as well as the presence of ANA-HEp2 antibodies (1:320 fine speckled type). Histopathological examination of the skin section revealed moderate perivascular infiltrates composed of lymphoid cells visible under the unaltered epidermis, without any diagnostic specificity. Direct immunofluorescence study on the skin biopsy did not reveal the presence of IgM, IgG, IgA, C3, and fibrinogen deposits. Simultaneously, a blood culture was obtained to investigate a potential infectious etiology due to the presence of fever and elevated procalcitonin levels. The culture yielded positive results for *Streptobacillus moniliformis*, a gram-negative bacterial species. The patient then disclosed his prior employment in rodent extermination.

**Table 1:** Results of the patient’s laboratory workup

	Results on admission	Results post-treatment	Reference ranges
White blood count [G/l]	20.5	5.64	4–10
Neutrophils [G/l]	19.04	3.35	2–7
C-reactive protein (CRP) [mg/l]	265.7	61	< 5
Procalcitonin [ng/ml]	6.31	–	< 0.5
Creatinine [mg/dl]	1.04	1.03	0.7–1.3
Glomerular filtration rate [ml/min/1.73 m^2^]	> 90	> 90	> 60
Alanine aminotransferase [U/l]	32	15	< 55
Aspartate aminotransferase [U/l]	58	23	5–34
Red blood cells in urine sample [FOV]	0–3	–	< 3
Protein in urine sample [G/l]	0.43	–	None
Rheumatoid factor (RF) [IU/ml]	< 20	–	< 30
Anti-cyclic citrullinated peptide antibodies [U/ml]	0.4	–	< 5
Anti-neutrophil cytoplasmic antibodies (ANCA)	1:1280 (cANCA/pANCA)	–	Negative
Antinuclear antibodies (ANA-HEp2)	1:320 (fine speckled type)	–	Negative
Anti-endothelial cell antibodies (AECA)	1:2560	–	Negative
Complement component C3 [G/l]	1.31	–	0.9–1.8
Complement component C4 [G/l]	0.24	–	0.1–0.4
Protein concentration in the cerebrospinal fluid [G/l]	0.48	–	0.15–0.4

Since the admission to the hospital, the patient was treated with antibiotics, initially with ceftriaxone and vancomycin. Subsequently, ciprofloxacin was added due to unsatisfactory improvement in the patient's general condition and episodes of increased body temperature. Non-steroidal anti-inflammatory drugs were used for arthritis.

Once the blood culture was completed, the antibiotic therapy was modified based on the antibiogram, with ampicillin being added and ceftriaxone and vancomycin being discontinued. As a result of the applied treatment the patient’s general condition improved, and the fever, as well as skin lesions and arthritis symptoms, gradually subsided. Laboratory tests showed a decrease in inflammatory markers, and liver and pancreatic enzymes.

After a thorough evaluation, the patient was diagnosed with rat-bite fever based on positive blood culture, history of employment in rodent extermination, and resolution of symptoms following antibiotic therapy without the need for immunosuppressive therapy. Due to the homelessness of the patient, it was not possible to follow up and verify the immunological tests.

## Search strategy

**Fig. 6:**
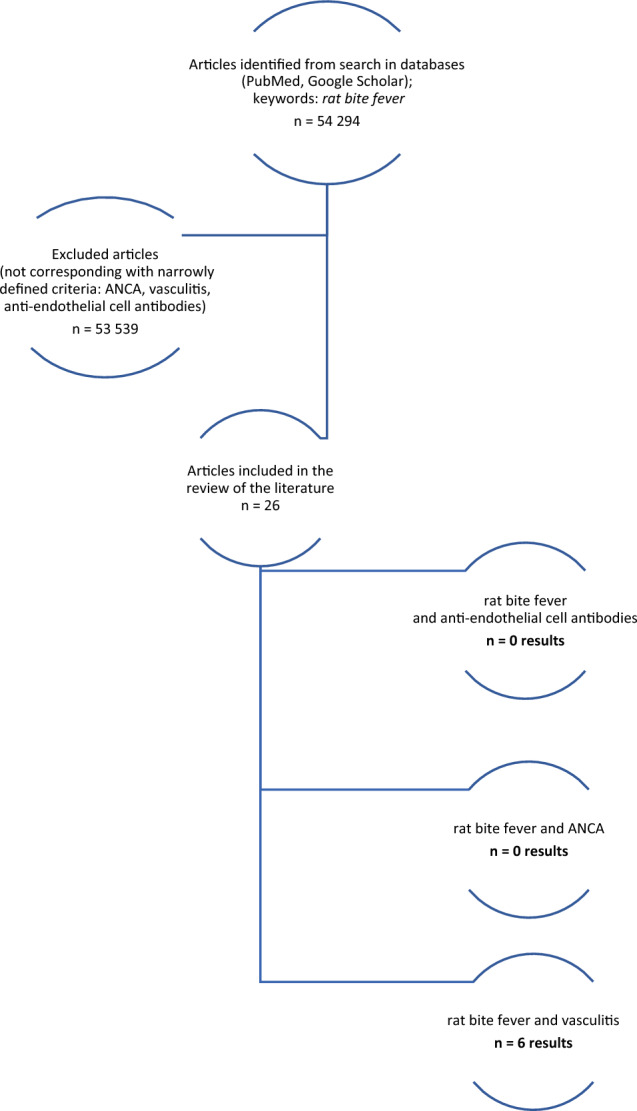
Flowchart presenting the results of the literature review

We have reviewed the English language literature for cases of RBF presenting with symptoms of vasculitis and/or antibody presence. The literature review was performed in PubMed and Google using the keywords “rat bite fever” AND “vasculitis”, “systemic vasculitis”, “ANCA”, “antiendothelial antibodies”. No time limits were applied. The flow chart is presented in Fig. 6.

We identified six cases of rat-bite fever in which the presence of accompanying signs of vasculitis was noted. Based on our literature review, two cases of systemic vasculitis were found to be associated with RBF, along with an additional four cases describing leukocytoclastic vasculitis in association with *S. moniliformis* infection. The cases are summarized in Table 2. Our review of published cases of rat-bite fever did not reveal any instances of ANCA antibodies being present. Several reports have indicated ANA positivity in cases of rat-bite fever, however, the titer levels were never found to be significantly high [9].

**Table 2:** Cases of RBF associated with vasculitis

Author	Age, sex	Clinical presentation	ANA positivity	ANCA positivity	Treatment	Outcome
Tattersall [10]	56, male	Fever, cough, sore throat, loose feces; acute polyarthritis affecting the right wrist, left thumb, both feet, and the right ankle; maculopapular, non-blanching rash over elbows, digits, and feet	No	No	Doxycycline	Significant improvement
Kawakami [11]	71, male	Fever, tachycardia, tachypnoë; polyarthralgia involving sternoclavicular joints, right wrist joint, and MCP joints of his right index and digitus medius; purpura and petechiae on the lower legs including the dorsum of the feet, accompanied by livedoid change on the lower legs and the upper legs	No	No	Ceftriaxone and azithromycin hydrate followed by amoxicillin hydrate	Complete resolution
Prouty [12]	9, male	Cough, cervical lymphangitis; edema of the extremities and face; red, macular rash on the lower extremities and the lower arms; arthralgia	No data	No data	Procaine penicillin	Death
Wilkins [13]	39, male	Fever, sore throat; macular rash on the whole body; pain in his right ankle, elbow and knee joints	No data	No data	Penicillin	Complete resolution
Albedwawi [14]	14, male	Fever; polyarthritis in shoulder, wrist, knee and ankle joints; petechial-purpuric rash over both lower limbs	No data	No data	Oral penicillin	Complete resolution
Tandon [15]	24, female	Fever, headache; erythematous to dusky purple skin papules on hands and feet; asymmetric migratory arthralgias involving the small joints of the extremities and, the knee, elbow, and shoulder joints	ANA 1:320	No data	No data	No data

## Discussion

The review of published cases of RBF revealed that the disease often manifests with migratory polyarthritis [6, 8] and purpura [7]. Due to the combination of symptoms reported by the patient, the differential diagnosis of the presented case posed a challenge. Until the blood culture analysis was finalized, the clinical presentation was consistent with a possible connective tissue disease. The differential diagnosis of joint and skin manifestation encompassed rheumatoid arthritis (RA) with vasculitis, reactive arthritis, and primary systemic vasculitis. The sudden onset of symptoms, positive procalcitonin, and absence of rheumatoid factor (RF) and anti-citrullinated protein antibody (ACPA) did not conform to the typical presentation of rheumatoid arthritis. The evaluation based on the ACR/EULAR 2010 classification criteria for rheumatoid arthritis resulted in a total score of three, comprising two points for joint involvement and one point for elevated inflammatory markers, which is insufficient for the diagnosis of RA. When formulating a differential diagnosis for a febrile patient presenting with seronegative arthritis, both infection-related arthritis and reactive arthritis should be considered. However, the absence of clinical manifestations, such as enteritis, urethritis, eye involvement (conjunctivitis or uveitis), as well as dactylitis, toe pain, and heel pain, speaks against a diagnosis of reactive arthritis.

The skin lesions were neither mucosal ulcers, keratoderma blennorrhagica, nor erythema nodosum. The clinical manifestation of arthritis coupled with specific cutaneous alterations and fever was more indicative of vasculitis. The observation of a high titer of ANCA strongly suggests an autoimmune background of the disease. However, it is important to note that while systemic ANCA-associated vasculitides are frequently accompanied by respiratory and urinary tract involvement, this was not observed in the presented case. Taken together, absence of dyspnea, cough, or hemoptysis in the patient's medical history, as well as the lack of interstitial changes in the lungs revealed by the chest CT scan, indicated no respiratory involvement.

There was also no evidence of renal impairment as indicated by the absence of hematuria or significant proteinuria in urine analysis.

Finally, the most crucial factor in differential diagnosis in the presented case proved to be the reduction of symptoms in response to antibiotic treatment, without the need for immunosuppressive therapy.

ANCAs are IgG antibodies that can be detected in most cases of ANCA-associated vasculitis (AAV). They target antigens in neutrophil granules and monocytes, predominantly myeloperoxidase (MPO)—pANCA or proteinase-3 (PR3)—cANCA (these two have been proven to be clinically significant [16]. ANCA of specificity other than PR3 and MPO can be detected by ELISA screening for human neutrophil elastase, lysosomal membrane protein-2 (LAMP-2-ANCA), bactericidal/permeability-increasing protein (BPI-ANCA), cathepsin G, lactoferrin, lysozyme, and other. It is believed that these ANCA specificities have no diagnostic value for patients with AAV and are sometimes mistakenly interpreted as clinically relevant. Patients with ANCA presence should receive a careful workup, because, apart from AAV, ANCA presence has been detected in a variety of other disorders, for example in patients suffering from inflammatory bowel disease (IBD), autoimmune liver disease, other connective tissue diseases (lupus, rheumatoid arthritis, and scleroderma), drug-induced vasculitis, and infections [17]. Various viral, bacterial, fungal, or protozoal infections can mimic AAV. It has also been proven that infections have been implicated as a trigger for ANCA production, AAV development or relapses. The formation of ANCA in our patient is likely to have been induced by a *Streptobacillus moniliformis* infection.

Infectious agents can induce vasculitis through various direct and indirect mechanisms [18]. The most frequent mechanisms include the immune response caused by vascular wall damage (direct, for example, rickettsial infection), humoral immune response with immune complex formation and deposition, molecular mimicry (autoantibody production, activation of autoreactive lymphocytes), and cell-mediated immune response with or without granulomata formation (indirect). Less commonly observed mechanisms involve infection-triggered immune dysregulation and anti-idiotypic response [19, 20]. It remains unclear whether leukocytoclastic vasculitis associated with *S. moniliformis* infection is caused by direct infection or indirect immune-mediated mechanisms.

Limited reports exist on infections inducing anti-neutrophil cytoplasmic antibody (ANCA)-associated vasculitis (AAV). The postulated mechanisms by which infections may trigger the generation of ANCA include autoantigen complementarity, epigenetic silencing, molecular mimicry, neutrophil extracellular traps (NETs) formation, and NETosis [21]. The conditions most frequently associated with vasculitis are infective endocarditis, caused by *Staphylococcus aureus*, and tuberculosis [22]. In tropical countries, tuberculosis, leprosy, and malaria can produce ANCA positivity in about 20–30% of cases [23]. Moreover, many cases of ANCA-associated vasculitis developed after COVID-19 have been recently reported [24–26]. Since the clinical manifestations of AAV can mimic those caused by various infectious agents, it is essential to identify the pathogens capable of inducing AAV. Therefore, the first step in the clinical approach to cutaneous vasculitis is to exclude infection-induced vasculitis [27]. Autoantibodies specifically targeting endothelial cell antigens (AECA) have also been detected in a different group of patients with small vasculitis, although they are not pathognomonic for any specific disease.

## Conclusion

Rat-bite fever is an uncommon bacterial disease transmitted by rats. Its symptoms are nonspecific and may include general weakness, intermittent fever, leukocytoclastic vasculitis, and arthritis. It may also present with ANCA production, as reported in this case study, which, to our knowledge, is the first to associate ANCA positivity with RBF. A definitive diagnosis of RBF can be made upon the result of blood cultures, which should show the presence of *Streptobacillus moniliformis* or *Spirillum minus*. It is important to note that many infectious diseases, such as infective endocarditis caused by *Staphylococcus aureus* and tuberculosis, can mimic AAV. Therefore, in the clinical approach to cutaneous vasculitis, it is crucial to first exclude infection-induced vasculitis.

## Data Availability

The data used and analysed during the current study are available from the corresponding author on reasonable request.

## References

[1] Gaastra W, Boot R, Ho HTK, Lipman LJA (2009). Rat bite fever. Vet Microbiol.

[2] Gupta M, Bhansali RK, Nagalli S, Oliver TI (2023). Rat-bite fever.

[3] Giorgiutti S, Lefebvre N (2019). Rat bite fever. N Engl J Med.

[4] Elliott SP (2007). Rat bite fever and *Streptobacillus **moniliformis*. Clin Microbiol Rev.

[5] Legout L, Senneville E, Mulleman D, Solau-Gervais E, Flipo RM, Mouton Y (2005). Rat bite fever mimicking rheumatoid arthritis. Scand J Infect Dis.

[6] Kache PA, Person MK, Seeman SM, McQuiston JR, McCollum J, Traxler RM (2020). Rat-bite fever in the United States: an analysis using multiple national data sources. Open Forum Infect Dis.

[7] Kasuga K, Sako M, Kasai S, Yoshimoto H, Iihara K, Miura H (2018). Rat bite fever caused by *Streptobacillus **moniliformis* in a cirrhotic patient initially presenting with various systemic features resembling Henoch-Schönlein Purpura. Intern Med.

[8] Wang TK, Wong SS (2007). *Streptobacillus **moniliformis* septic arthritis: a clinical entity distinct from rat-bite fever?. BMC Infect Dis.

[9] Akter R, Boland P, Daley P, Rahman P, Al Ghanim N (2016). Rat bite fever resembling rheumatoid arthritis. Can J Infect Dis Med Microbiol.

[10] Tattersall RS, Bourne JT (2003). Systemic vasculitis following an unreported rat bite. Ann Rheum Dis.

[11] Kawakami Y, Katayama T, Kishida M, Oda W, Inoue Y (2016). A case of *Streptobacillus moniliformis* infection with cutaneous leukocytoclastic vasculitis. Acta Med Okayama.

[12] Prouty M, Schafer EL (1950). Periarteritis nodosa associated with rat bite fever due to *Streptobacillus **moniliformis* (erythema arthriticum epidemicum). J Pediatr.

[13] Wilkins EG, Millar JG, Cockcroft PM, Okubadejo OA (1988). Rat-bite fever in a gerbil breeder. J Infect.

[14] Albedwawi S, LeBlanc C, Shaw A, Slinger RW (2006). A teenager with fever, rash and arthritis. CMAJ.

[15] Tandon R, Lee M, Curran E, Demierre MF, Sulis CA (2006). A 26-year-old woman with a rash on her extremities. Clin Infect Dis.

[16] Kronbichler A, Lee KH, Denicolò S, Choi D, Lee H, Ahn D, Kim KH, Lee JH, Kim H, Hwang M, Jung SW, Lee C, Lee H, Sung H, Lee D, Hwang J, Kim S, Hwang I, Kim DY, Kim HJ, Cho G, Cho Y, Kim D, Choi M, Park J, Park J, Tizaoui K, Li H, Smith L, Koyanagi A, Jacob L, Gauckler P, Shin JI (2020). Immunopathogenesis of ANCA-associated vasculitis. Int J Mol Sci.

[17] Moiseev S, Cohen Tervaert JW, Arimurac Y, Bogdanos DP (2020). 2020 international consensus on ANCA testing beyond systemic vasculitis. Autoimmunity Rev.

[18] Yang L, Xie H, Liu Z (2018). Risk factors for infectious complications of ANCA-associated vasculitis: a cohort study. BMC Nephrol.

[19] Pipitone N, Salvarani C (2008). The role of infectious agents in the pathogenesis of vasculitis. Best Pract Res Clin Rheumatol.

[20] Satta R, Biondi G (2015). Vasculitis and infectious diseases. G Ital Dermatol Venereol.

[21] Konstantinov KN, Ulff-Møller CJ, Tzamaloukas AH (2015). Infections and antineutrophil cytoplasmic antibodies: triggering mechanisms. Autoimmun Rev.

[22] Kakoullis L, Parperis K, Papachristodoulou E, Panos G (2020). Infection-induced myeloperoxidase specific antineutrophil cytoplasmic antibody (MPO-ANCA) associated vasculitis: a systematic review. Clin Immunol.

[23] Izci Duran T, Turkmen E, Dilek M, Sayarlioglu H, Arik N (2021). ANCA-associated vasculitis after COVID-19. Rheumatol Int.

[24] Morris D, Patel K, Rahimi O, Sanyurah O, Iardino A, Khan N (2021) ANCA vasculitis: A manifestation of Post-Covid-19 Syndrome. Respir Med Case Rep 34:101549. 10.1016/j.rmcr.2021.101549 10.1016/j.rmcr.2021.101549 PMC8580553 34786334

[25] Becker RC (2020). COVID-19-associated vasculitis and vasculopathy. J Thromb Thrombolysis.

[26] Ghosh K, Pradhan V, Ghosh K (2008). Background noise of infection for using ANCA as a diagnostic tool for vasculitis in tropical and developing countries. Parasitol Res.

[27] Chen KR, Carlson JA (2008). Clinical approach to cutaneous vasculitis. Am J Clin Dermatol.

